# Structural and Functional Retinal Changes in Patients with Mild Cognitive Impairment with and without Diabetes

**DOI:** 10.3390/jcm12227035

**Published:** 2023-11-10

**Authors:** Álvaro Santos-Ortega, Carmen Alba-Linero, Facundo Urbinati, Carlos Rocha-de-Lossada, Rafael Orti, José Antonio Reyes-Bueno, Francisco Javier Garzón-Maldonado, Vicente Serrano, Carmen de Rojas-Leal, Carlos de la Cruz-Cosme, Manuela España-Contreras, Marina Rodríguez-Calvo-de-Mora, Natalia García-Casares

**Affiliations:** 1 Department of Ophthalmology, Hospital Costa del Sol, 29603 Malaga, Spain; alvaro.santos.sspa@juntadeandalucia.es; 2 Department of Ophthalmology, Hospital Universitario Virgen de la Victoria, 29010 Malaga, Spain; 3 Department of Ophthalmology, Faculty of Medicine, University of Malaga, 29016 Malaga, Spain; rortigarcia@gmail.com; 4 Department of Ophthalmology, Hospital Regional Universitario, 29011 Malaga, Spain; facundo.urbinati.sspa@juntadeandalucia.es (F.U.); crocha1@us.es (C.R.-d.-L.); manuela.espana.sspa@juntadeandalucia.es (M.E.-C.); marina.rodriguez.calvomora.sspa@juntadeandalucia.es (M.R.-C.-d.-M.); 5 Qvision, Opththalmology Department, VITHAS Almería Hospital, 04120 Almería, Spain; 6 Ophthalmology Department, VITHAS Málaga, 29016 Malaga, Spain; 7 Department of Surgery, Faculty of Medicine, Ophthalmology Area Doctor Fedriani, University of Sevilla, 41004 Sevilla, Spain; 8 Department of Neurology, Hospital Regional Universitario, 29011 Malaga, Spain; jantonio.reyes.sspa@juntadeandalucia.es; 9 Department of Neurology, Hospital Virgen de la Victoria, 29010 Malaga, Spain; franciscoj.garzon.sspa@juntadeandalucia.es (F.J.G.-M.); vicente.serrano.sspa@juntadeandalucia.es (V.S.); carmen.rojas.leal.sspa@juntadeandalucia.es (C.d.R.-L.); carlos.cruz.sspa@juntadeandalucia.es (C.d.l.C.-C.); 10 Instituto de Investigación Biomédica de Málaga (IBIMA), 29010 Malaga, Spain; nagcasares@uma.es; 11 Department of Medicine, Faculty of Medicine, University of Malaga, 29016 Malaga, Spain; 12 Centro de Investigaciones Médico-Sanitarias (CIMES), University of Malaga, 29016 Malaga, Spain

**Keywords:** Alzheimer’s disease, mild cognitive impairment, diabetes, optical coherence tomography angiography

## Abstract

Our objective is to analyze retinal changes using optical coherence tomography angiography (OCT-A) in patients with mild cognitive impairment (MCI) to characterize structural and vascular alterations. This cross-sectional study involved 117 eyes: 39 eyes from patients with MCI plus diabetes (DM-MCI), 39 eyes from patients with MCI but no diabetes (MCI); and 39 healthy control eyes (C). All patients underwent a visual acuity measurement, a structural OCT, an OCT-A, and a neuropsychological examination. Our study showed a thinning of retinal nerve fiber layer thickness (RNFL) and a decrease in macular thickness when comparing the MCI-DM group to the C group (*p* = 0.008 and *p* = 0.016, respectively). In addition, an increase in arteriolar thickness (*p* = 0.016), a reduction in superficial capillary plexus density (*p* = 0.002), and a decrease in ganglion cell thickness (*p* = 0.027) were found when comparing the MCI-DM group with the MCI group. Diabetes may exacerbate retinal vascular changes when combined with mild cognitive impairment.

## 1. Introduction

Alzheimer’s disease (AD) is the most frequent cause of cognitive impairment and dementia in persons over 65 years of age around the world, as well as being an important cause of death in many countries [1]. The prevalence of AD is increasing. Such is its importance that it is estimated that by 2050 its prevalence in the United States will have tripled as a result of population growth and increased life expectancy [2]. 

AD could be defined as a continuous process which initiates decades before overt cognitive impairment [3,4].

Mild cognitive impairment (MCI) is the long prodromal stage in which the presence of symptoms that are so non-specific that they rarely lead to a diagnosis (apathy, anxiety, etc.) stands out [5]. The disease will evolve over time. Patients’ symptoms are heterogeneous, with mood disorders, sleep disorders, hallucinations, apraxia, agnosia, or confusion [2]. 

An important challenge is to obtain an efficient tool allowing us to achieve an early diagnosis in patients with MCI, which could help slow down its progression and avoid reaching the stage of dementia [6,7].

MCI is currently mainly identified by cognitive function testing and clinical evaluation. Cerebrospinal fluid analyses (tau and amyloid proteins), neuroimaging and positron emission tomography scans that can provide a more objective analysis are invasive, expensive, and have not been widely used in daily clinical practice [8,9]. The techniques for screening for the disease focus on neurocognitive tests: the Minimental State Examination (MMSE) and the Montreal Cognitive Assessment (MoCA), as they are the fastest and most efficient [6].

Microvascular changes in Alzheimer patients play a critical role in contributing to the occurrence and progression of the disease and can be found even in preclinical stages and serve as biomarkers [10,11,12,13,14].

Based on the fact that the retina has the same embryological development as the brain, and shares functional and anatomical features, several researchers have considered whether it would be possible to detect cerebral diseases at an early stage by studying the retina and certain intraocular biomarkers related to its trophism [15,16,17]. 

Through optical coherence tomography (OCT), we have the capability to identify and quantify early changes in retinal layer thickness, serving as a reliable marker of axonal damage or neuroinflammation that manifest in neurodegenerative diseases [18].

The first study to use optical coherence tomography angiography (OCT-A) to evaluate the vascular changes in AD patients was done by Bulut et al. [19]. They found a reduced vessel density in AD that was correlated with worse scores in cognitive tests. 

OCT-A is a fast, safe, and efficient technique. It has a great spatial definition, obtaining images and measurements with micrometric precision, in addition to which it is harmlessness for patients and its cost is low [15,20]. OCT-A can provide a quantitative evaluation of microvascular perfusion and can be used to measure the density of the superficial retinal capillary plexuses (SRCP), the deep retinal capillary plexuses (DRCP), and the area of the foveal avascular zone.

Diabetes has significant adverse effects on blood vessels, leading to an augmented risk of small cerebral vessel disease [21]. Type 2 diabetes mellitus (DM2) is associated with an increased risk of MCI of up to 60% [22] and its worsening and progression to dementia [23]. Furthermore, experimental studies carried out on Ins2^Akita^ diabetic mice provide evidence and support the significance of retinal alterations that manifest in diabetes for comprehending potential pharmacological interventions [24]. 

The retina of patients with DM2 may show functional and structural impairments, preceding the clinical manifestation of diabetic retinopathy [25]. 

The objective of this study was to determine the possibility of reaching an early diagnosis of AD based on trophic changes produced in the retina [26]. Our study also included DM2 patients, who have a worse prognosis in both retinal and cognitive changes, as has been shown in multiple studies [26,27].

## 2. Materials and Methods

This cross-sectional study analyzed the changes in retinal vascularization and nerve fiber thickness in association with the cognitive deterioration presented by the patients, as well as the possible influence of diabetes on both variables. The study was carried out jointly at the Regional University Hospital of Malaga (HRUM), the University Clinical Hospital of Malaga (HUVV), and the Medical-Health Research Center (CIMES).

A total of 120 eyes, corresponding to both eyes of 60 patients, were analyzed. Three eyes were excluded due to the presence of cataracts, resulting in a total of 117 eyes, of which 74 had undergone cataract surgery, with no significant differences between the groups. In the remaining 43 eyes, the lens was either transparent or exhibited a degree of lens sclerosis without clinical significance.

Patients with DM2 were selected through endocrinology services, and patients with MCI were selected from neurology services. Group 1 comprised 39 eyes of 20 patients with MCI and DM2 (DM-MCI), Group 2 comprised 39 eyes of 20 patients with MCI without DM2 (MCI), and Group 3 comprised 39 eyes of 20 healthy control subjects (C). 

Cognitive impairment was determined using the 2006 European Alzheimer’s Disease Consortium criteria, including memory complaints, a MoCA score < 26, or a MMSE score < 24, clinical dementia ≥ 0.5, and impaired activities of daily living.

Patients who signed the informed consent, were aged between 62 and 86 years, and for whom a complete follow-up was possible were included. Good visual acuity was an inclusion criterion. 

Patients were excluded if they had a history of chronic medical illness or a neurological condition that could affect cognitive function, head trauma with loss of consciousness, learning disorder or intellectual disability, a documented history of cardiovascular disease, active cancer or a history of malignant tumor in the last 5 years, serious psychiatric disorders, a history of ocular inflammatory disease, alterations of the optic disc, opacification preventing correct visualization of the eye, extremely low visual acuity (<0.1), age-related macular degeneration, or a history of eye surgery (except cataract extraction). 

The differential criterion (DM2) between Groups 1 and 2 was diagnosed according to the 2006 ADA criteria and the existence of diabetic retinopathy was ruled out by funduscopic examination. If DM2 was confirmed and the presence of diabetic retinopathy was ruled out, the patients were classified into Group 1, with the non-diabetic patients classified as Group 2.

Visual acuity (VA) was measured using a Snellen chart.

HD-OCT (Cirrus 5000) was used to quantify structural retinal changes as peripapillary arteriole and venule calibers, total and sectorial thickness of the ganglion cell layer (GCC), retinal nerve fiber layer thickness (RNFL), central and sectorial macular thickness, and macular volume. Qualitative and/or quantitative evaluation of the superficial and deep capillary plexus and foveal avascular zone were measured using Cirrus HD-OCT 500 with AngioPlex software version 11.5.2.54532 (acquisition protocol 3 × 3 mm).

A complete neuropsychological evaluation was performed including educational level and MMSE (mini-mental state examination).

### 2.1. Statistical Analyses

Qualitative variables were described using absolute frequencies and percentages. The description of quantitative variables was performed using mean and standard deviation (SD). The Kolmogorov–Smirnov test was used to assess the normality of the distributions. Student’s *t*-test and the Mann–Whitney test were performed in order to analyze the quantitative outcomes; the Welch test was used when there was no homoscedasticity. The results were described with *p*-values. For all the tests, *p*-values > 0.05 were considered statistically significant. Statistical analysis was performed using JASP Team (2022) software; JASP (Version 0.16.1) 

### 2.2. Ethical Considerations

This research was carried out under conditions of respect for the fundamental rights of the person and the ethical postulates that affect biomedical research with human beings following, for these purposes, the international recommendations contained in the Declaration of Helsinki and its subsequent revisions. The treatment of personal data was carried out in accordance with current regulations, RD 223/2004 of February 6 and Organic Law 15/1999 of December 13 on the protection of personal data.

## 3. Results

Our study showed an increase in arteriolar thickness in the DM-MCI group when compared to the C group (*p* = 0.016), along with a decrease in central superficial capillary plexus density (*p* = 0.002) when comparing the DM-MCI group to the C group. The optic nerve fiber density and macular thickness were also lower when comparing patients from the DM-MCI group with group C (*p* < 0.05). In addition, a tendency for a decrease in the ganglion cell layer thickness was also observed in the DM-MCI group compared to the MCI group, mainly at the superior nasal (*p* = 0.27) and inferior nasal (*p* = 0.027) quadrant. These data are presented in Table 1, Table 2, Table 3, Table 4 and Table 5.

### 3.1. Demographic and Questionnaire Data

All 60 people in the study were Caucasian, with 32 men and 28 women. The distribution by groups was as follows: 15 men and 5 women in the DM-MCI group, 8 men and 12 women in the MCI group, and 9 men and 11 women in the C group.

The mean age of the patients was 72.27 years: DM-MCI, 72.65 years, MCI, 73.72 years; C, 70.44 years (*p* = 0.094).

The mean MMSE scores were as follows: DM-MCI, 26.93; MCI, 27.23; C, 28.44 with statistically significant differences between the groups with DM-MCI and MCI compared to the control group (*p* = 0.034) but not between DM-MCI and MCI, as shown in Table 1.

### 3.2. Visual Acuity Data

In our study no significant differences were found in relation to VA when comparing between the groups. 

The mean VA expressed on a decimal scale in the DM-MCI group was 20/25 on the Snellen scale, in the MCI group it was 20/20 on the Snellen scale, and in the C group it was 20/25 on the Snellen scale (Table 1).

### 3.3. Optical Coherence Tomography Angiography Data (OCT-A)

#### 3.3.1. Vascular Parameters

An increase in arteriolar caliber was observed in the DM-MCI group in relation to the MCI group (*p* = 0.016), but which was absent in the comparison between the DM-MCI and C (*p* = 0.1) and MCI and C (*p* = 0.761) groups.

A decrease in the central superficial capillary plexus (SCP) was observed in the DM-MCI group compared to the MCI group (*p* = 0.002) and no differences were observed in the DM-MCI group compared to the C group t(*p* = 0.055) and the MCI group and the C group (*p* = 0.882).

**Figure 1:**
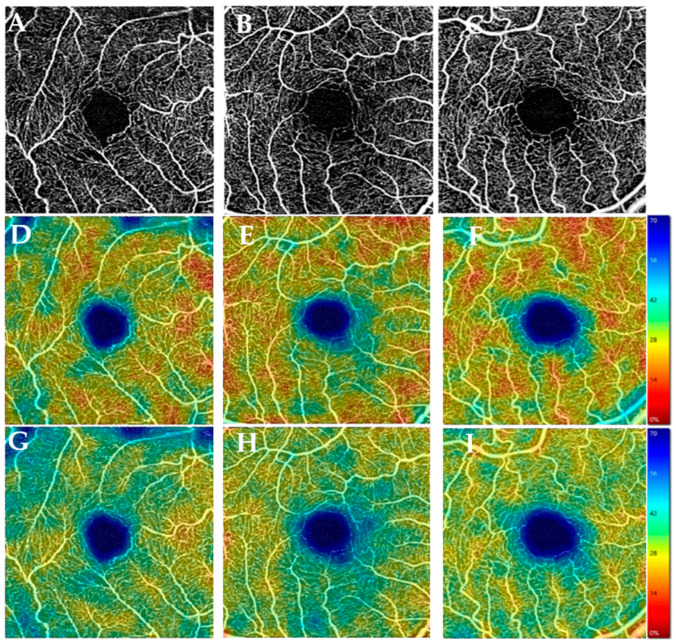
Optical coherence tomography angiography (3 × 3 mm images). Superficial capillary plexus analysis using Angioplex OCTA software 11.5.2.54532 (Carl Zeiss Meditec, Dublin, CA) from a DM-MCI patient (**A**), MCI patient (**B**), and control (**C**) and their corresponding quantitative color maps of vessel density (**D**–**F**) and perfusion density (**G**–**I**) with the scale on the right. MCI group = patients with mild cognitive impairment without diabetes. C group = healthy patients.

Regarding the size of the foveal avascular zone, in our study, we found no significant differences between the DM-MCI, MCI, and C groups (Figure 1). These data are represented in Table 2.

#### 3.3.2. Structural Parameters

**Figure 2:**
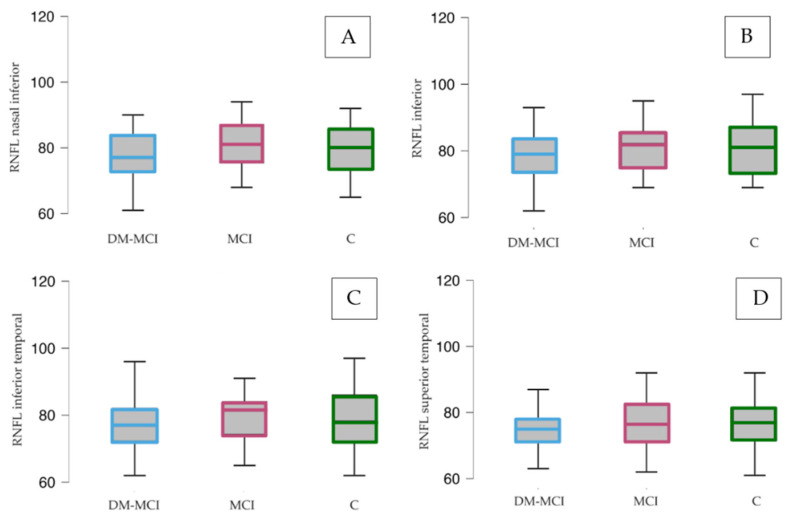
RNFL thickness analysis. RNFL thickness (expressed in µm) in nasal inferior (**A**), inferior (**B**), temporal inferior (**C**), and temporal superior (**D**) quadrants. RNFL = retinal nerve fiber layer. DM-MCI group = patients with mild cognitive impairment and diabetes. MCI group = patients with mild cognitive impairment without diabetes. C group = healthy patients.

We observed a generalized decrease in optic nerve fiber density when comparing patients from the DM-MCI group with the C group (*p* < 0.05), except the upper quadrant and mean value, where the differences were not statistically significant (*p* = 0.42 and *p* = 0.53, respectively). See Table 3 and Figure 2.

A tendency for a decrease in the ganglion cell layer thickness was also observed in the DM-MCI group compared to the MCI group, mainly at the superior quadrant (*p* = 0.02). This effect was not observed when comparing the DM-MCI group with the C group, nor in the MCI group compared to C group patients. These data can be assessed in Table 4.

**Figure 3:**
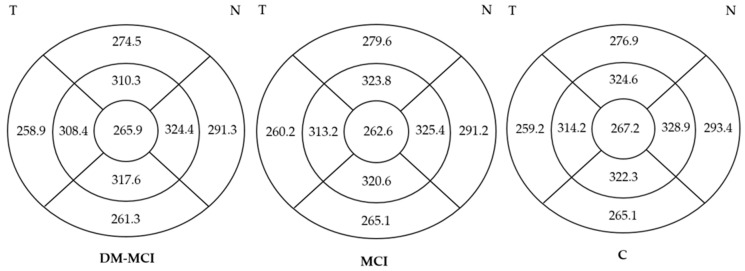
Comparison of macular thickness in DM-MCI, MCI, and C groups. A generalized decrease is observed in the DM-MCI group compared to the C group. Inner ring = 1 × 1. Inner ring = 3 × 3. Outer ring = 6 × 6. DM-MCI group = patients with mild cognitive impairment and diabetes. MCI group = patients with mild cognitive impairment without diabetes. C group = healthy patients.

**Figure 4:**
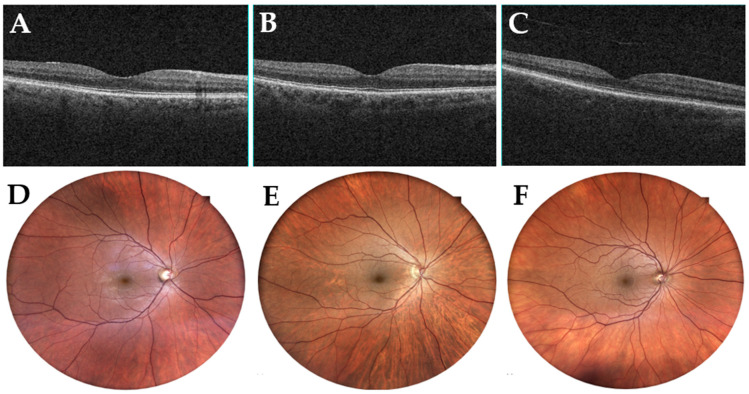
Examples of structural optical coherence tomography and fundus images. Macular OCT of a patient from the DM-MCI group, MCI group, and C group (**A**–**C**) and their corresponding fundus images (**D**–**F**). DM-MCI group = patients with mild cognitive impairment and diabetes. MCI group = patients with mild cognitive impairment without diabetes. C group = healthy patients.

Analysis of the macular thickness showed a generalized decrease, in all quadrants, in patients in the DM-MCI group compared to the C group, mainly in inner sectors (*p* < 0.05). No significant differences were observed when comparing the DM-MCI group to the MCI group, nor when comparing the MCI group to the C group (Figure 3 and Figure 4). These data are presented in Table 5.

## 4. Discussion

In Alzheimer’s disease (AD), increasingly prevalent due to increased life expectancy, histological and microvascular changes can be observed, more evident the more advanced the disease [13,19,28]. However, early diagnosis, even in the preclinical phase, is crucial for the proper management and follow-up of these patients.

Advances in current exploration techniques, e.g., structural OCT and OCT-A, have enabled detailed and precise study of the structural and microvascular changes of the retina and the optic nerve as a projection of the central nervous system, present even in the preclinical stages of patients with cognitive impairment [18,29]. 

However, other diseases with microvascular involvement, such as diabetes mellitus, can coexist in a patient with cognitive impairment, and the combined effect of both diseases on the microvasculature and retinal parenchyma is still poorly understood [21]. 

To the best of our knowledge, this is the first study that assesses these two conditions.

### 4.1. Microvasculature

In our study, in relation to microvasculature, a significant decrease was observed in SCP in the DM-MCI group when compared to the control group, with no significant differences in the other groups. There were also no significant differences found in the FAZ.

Most studies in the literature have shown that patients with AD exhibit a reduced SCP and an increase in the FAZ [13,19,29,30,31]. Nevertheless, when analyzing preclinical stages such as MCI, findings in microvasculature can be contradictory, as studies present both non-significant results [13,28,31], a decrease [19,29,32], and even an increase in SCP, along with FAZ size not proportional to vascular density [16]. One hypothesis to explain these contradictory findings may be attributed to the different stages of the disease, as in very early stages, there might be an inflammatory component due to amyloid accumulation [33], leading to hypoxia and favoring an increase in SCP flow [34]. However, in later stages, the damage incurred results in a decrease in vascular density and an increase in FAZ size [13,19,35]. Therefore, the role of microvasculature in the preclinical stages of neurodegenerative diseases like MCI remains uncertain and requires further investigation. 

One of the advantages of this study lies in the relatively uniform age distribution among the patients, as it is well-documented that advanced age by itself can lead to a reduction in vascular density and an enlargement in FAZ [36].

It is well established that in diabetic patients, even without retinopathy, a decrease in SCP and an increase in the FAZ can be documented [37,38,39]. Consequently, this would explain why statistically significant differences are found in the DM-MCI group, unlike in the other groups. 

This study also observed an increase in arteriolar thickness at the level of the optic disc in patients with DM and MCI compared to the controls. These findings are consistent with several authors [16,40] who found no significant differences between MCI and healthy patients, although no diabetic patients were included in these studies. Here we highlight the importance of the effect of DM2, as in the DM2 group, an increase in arteriolar thickness was observed, although when individuals with cognitive impairment but without diabetes were compared with healthy individuals this was not evident, contrary to other studies [41]. 

This study found no significant changes in the thickness of the venule at the level of the papilla, so it could be said that its structure seems to not be affected by MCI or DM2.

### 4.2. Structural Parameters 

In relation to the ganglion cell layer (GCL) and retinal nerve fiber layer (RNFL) thickness, we observed a thinning of RNFL in the DM-MCI group compared to the controls, particularly in the lower sectors, and a decrease in the central macular GCL when comparing DM-MCI to MCI. These findings are consistent with other studies [42,43]. 

In established Alzheimer’s disease (AD), most studies consistently reveal a decrease in RNFL thickness—mainly in lower [44,45] and upper sectors [46,47]—and in GCL thickness [48,49]. Some of these alterations can also be observed in individuals with MCI [50,51,52]. Nevertheless, throughout the progression of AD, dynamic changes occur during its successive stages, and it is not uncommon to encounter inconclusive results in the early phases when the extent of the damage has not been definitively established [42]. On the other hand, recent studies have shown that there are no significant differences in retinal layer thickness between individuals in the preclinical stages of AD and control subjects [53]. 

Concerning macular thickness, within our sample, we observed a reduction in group A when compared to group C. Several studies propose that neurodegenerative diseases affect multiple retinal layers, including the inner plexiform layer (IPL), inner nuclear layer (INL), and outer plexiform layer (OPL), not limited to RNFL and CCG, since retinal cells comprise microglia and Müller cells, which can be influenced to exhibit inflammatory responses [53,54]. Additionally, most studies have focused on established AD, with only a limited number of studies delving into the inner and outer retinal layers in preclinical AD, leading to inconsistent findings. In the preclinical stages, there exists considerable variation in results, with studies demonstrating thickening [55], thinning [56], as well as others that do not reveal significant differences [53,57]. Likely, the pattern and degree of involvement of the retinal layers significantly rely on the stage, inclusion criteria, and interindividual variability, which largely remains unknown.

## 5. Conclusions

Ophthalmoscopic microvascular and structural findings in the preclinical stages of neurodegenerative diseases should be carefully evaluated. Due to the coexisting phenomena of hypoxia and inflammation accompanying initial damage in the early stages of MCI, values indicating both increases and decreases in vascular thickness and density may be encountered.

This should be especially taken into consideration when vascular diseases like diabetes mellitus coexist, as they can also alter these values and consequently lead to erroneously exacerbating the degree of anatomical involvement in these patients.

## Figures and Tables

**Table 1:** Demographic, visual acuity, and cognitive test data.

	DM-MCI Group	MCI Group	C Group	DM-MCI vs. MCI	DM-MCI vs. C	MCI vs. C
	*n* = 39	*n* = 39	*n* = 39	*n* = 78	*n* = 78	*n* = 78
	Mean (95% CI)			*p* Value		
%Gender (Male/Female)	15/5 (75/15)	8/12 (40/60)	9/11 (45/55)	0.130 ^b^	0.199 ^b^	0.421 ^b^
Age (years)	72,654 (74,201–71,107)	73,720 (75,490–71,950)	70,440 (71,445–69,435)	0.010 ^b^	0.061 ^b^	0.055 ^b^
Visual acuity (decimal)	0.840 (0.914–0.766)	0.905 (0.951–0.859)	0.856 (0.928–0.785)	0.199 ^b^	0.554 ^b^	0.308 ^b^
MMSE	26.93 (28.83–25.03)	27.23 (28.91–25.55)	28.44 (29.77–27.11)	0.420 ^a^	<0.001 ^a^	0.034 ^c^

^a^ = Student’s t; ^b^ = Mann–Whitney U; ^c^ = Welch test. MMSE = mini-mental state examination. DM-MCI group = patients with mild cognitive impairment and diabetes. MCI group = patients with mild cognitive impairment without diabetes. C group = healthy patients.

**Table 2:** Vascular analysis results.

	DM-MCI Group	MCI Group	C Group	*p* Value		
	*n* = 39	*n* = 39	*n* = 39	*n* = 78	*n* = 78	*n* = 78
	Mean (95% CI)			DM-MCI vs. MCI	DM-MCI vs. C	MCI vs. C
Peripapillary venule diameter	149.205 (140.50–157.91)	146.590 (139.699–153.481)	151.744 (144.236–159.252)	0.323 ^a^	0.667 ^a^	0.838 ^a^
Peripapillary arteriole diameter	121.744 (113.43–130.06)	110.026 (102.372–117.680)	114.256 (105.528–120.985)	0.016 ^b^	0.100 ^b^	0.761 ^a^
Central SCP	10.403 (9675–11.130)	8710 (7835–9586)	9466 (8590–10.342)	0.002 ^a^	0.055 ^a^	0.882 ^a^
Inner SCP	19.743 (19.050–20.437)	19.297 (18.367–20.228)	19.282 (18.634–19.929)	0.354 ^b^	0.119 ^b^	0.345 ^b^
Complete SCP	18.690 (18.034–19.345)	18.331 (17.645–19.017)	18.163 (17.522–18.804)	0.228 ^b^	0.112 ^b^	0.439 ^b^
FAZ area	0.233 (0.206–0.260)	0.278 (0.248–0.309)	0.253 (0.224–0.283)	0.983 ^a^	0.834 ^a^	0.129 ^a^
FAZ perimeter	2.142 (2.013–2.270)	2.402 (2.248–2.556)	2.249 (2.140–2.357)	0.994 ^a^	0.891 ^a^	0.058 ^b^
FAZ circularity	0.631 (0.602–0.659)	0.600 (0.564–0.637)	0.608 (0.578–0.639)	0.101 ^a^	0.151 ^a^	0.504 ^b^

^a^ = Student’s t; ^b^ = Mann–Whitney U. SCP: superficial capillary plexus. FAZ: foveal avascular zone. DM-MCI group = patients with mild cognitive impairment and diabetes. MCI group = patients with mild cognitive impairment without diabetes. C group = healthy patients.

**Table 3:** Structural parameter results: retinal nerve fiber layer thickness.

	DM-MCI Group	MCI Group	C Group	*p* Value		
	*n* = 39	*n* = 39	*n* = 39	*n* = 78	*n* = 78	*n* = 78
	Mean (95% CI)			DM-MCI vs. MCI	DM-MCI vs. C	MCI vs. C
Mean	77,513 (75,326–79,700)	78,500 (76,317–80,683)	76,282 (72,968–79,596)	0.533 ^a^	0.530 ^b^	0.706 ^b^
Temporal superior	27,897 (25,324–30,471)	29,711 (27,090–32,331)	31,897 (29,010–34,784)	0.321 ^b^	0.019 ^b^	0.140 ^b^
Superior	16,128 (14,537–17,719)	17,026 (15,566–18,487)	16,333 (14,807–17,860)	0.357 ^b^	0.428 ^a^	0.664 ^b^
Nasal inferior	25,769 (23,905–27,634)	26,763 (24,589–28,938)	28,463 (24,665–32,258)	0.173 ^b^	0.098 ^b^	0.222 ^b^
Nasal superior	34,872 (33,477–36,266)	37,105 (35,143–39,068)	38,897 (35,566–42,229)	0.099 ^b^	0.017 ^c^	0.184 ^c^
Inferior	35,897 (35,838–40,005)	37,921 (35,838–40,005)	40,692 (37,342–44,043)	0.142 ^a^	0.008 ^c^	0.087 ^c^
Temporal inferior	37,949 (36,017–39,881)	39,684 (37,666–41,702)	42,872 (38,428–45,316)	0.411 ^b^	0.028 ^c^	0.143 ^c^

^a^ = Student’s t; ^b^ = Mann–Whitney U; ^c^ = Welch test. RNFL: retinal nerve fiber layer thickness. DM-MCI group = patients with mild cognitive impairment and diabetes. MCI group = patients with mild cognitive impairment without diabetes. C group = healthy patients.

**Table 4:** Structural parameter results: ganglion cell layer thickness.

	DM-MCI Group	MCI Group	C Group	*p* Value		
	*n* = 39	*n* = 39	*n* = 39	*n* = 78	*n* = 78	*n* = 78
	Mean (95% CI)			DM-MCI vs. MCI	DM-MCI vs. C	MCI vs. C
Average	79.11 (73.81–84.41)	78.85 (72.41–85.21)	80.00 (72.62–87.38)	0.482 ^a^	0.396 ^a^	0.422 ^a^
Temporal superior	75.85 (70.24–81.47)	77.00 (70.73–83.27)	74.41 (59.80–89.63)	0.662 ^b^	0.726 ^c^	0.743 ^a^
Superior	79.07 (73.22–84.92)	79.80 (73.08–70.73)	83.10 (69.30–96.90)	0.029 ^a^	0.162 ^c^	0.398 ^a^
Nasal inferior	77.82 (71.86–83.78)	79.80 (72.35–87.25)	80.36 (73.60–87.11)	0.138 ^b^	0.124 ^a^	0.407 ^a^
Nasal superior	80.00 (75.25–85.75)	79.30 (72.16–86.44)	82.29 (71.49–93.08)	0.412 ^b^	0.270 ^c^	0.224 ^a^
Inferior	80.68 (74.16–87.20)	81.55 (69.87–92.23)	79.93 (67.32–92.54)	0.500 ^b^	0.556 ^b^	0.685 ^a^
Temporal inferior	79.18 (72.73–85.63)	78.85 (72.11–84.99)	79.64 (71.67–87.62)	0.391 ^a^	0.457 ^a^	0.451 ^a^

^a^ = Student’s t; ^b^ = Mann–Whitney U; ^c^ = Welch test. GCL: ganglion cell layer. DM-MCI group = patients with mild cognitive impairment and diabetes. MCI group = patients with mild cognitive impairment without diabetes. C group = healthy patients.

**Table 5:** Structural parameter results: macular thickness.

	DM-MCI Group	MCI Group	C Group	*p* Value		
	*n* = 39	*n* = 39	*n* = 39	*n* = 78	*n* = 78	*n* = 78
	Mean (95% CI)			DM-MCI vs. MCI	DM-MCI vs. C	MCI vs. C
Average	275.333 (271.165–279.502)	276.553 (271.507–281.598)	278.385 (273.492–283.277)	0.301 ^b^	0.100 ^b^	0.303 ^b^
Central	265.974 (259.919–272.030)	262.632 (254.687–270.576)	267.154 (259.653–274.655)	0.180 ^b^	0.406 ^a^	0.068 ^b^
Temporal inner	308.410 (303.842–312.979)	313.184 (308.385–317.983)	314.179 (309.035–319.324)	0.101 ^b^	0.052 ^a^	0.300 ^b^
Temporal outer	258.897 (254.681–263.114)	260.216 (256.564–263.869)	259.154 (255.105–263.203)	0.454 ^b^	0.352 ^b^	0.648 ^a^
Nasal inner	324.359 (319.479–329.239)	325.447 (320.383–330.512)	328.872 (323.360–334.384)	0.325 ^b^	0.039 ^b^	0.099 ^b^
Nasal outer	291.282 (285.948–296.616)	291.211 (286.628–295.793)	293.385 (287.080–299.689)	0.592 ^b^	0.144 ^b^	0.196 ^b^
Superior inner	310.282 (294.223–326.341)	323.789 (318.849–328.730)	324.590 (319.516–329.663)	0.111 ^b^	0.040 ^b^	0.372 ^b^
Superior outer	274.487 (267.902–281.072)	279.553 (273.285–285.821)	276.872 (271.325–282.418)	0.071 ^b^	0.121 ^b^	0.781 ^b^
Inferior inner	317.641 (312.996–322.286)	320.553 (316.364–324.741)	322.308 (315.972–328.643)	0.105 ^b^	0.047 ^b^	0.192 ^b^
Inferior outer	261.308 (256.609–266.007)	265.079 (259.824–270.334)	265.051 (259.469–270.634)	0.343 ^b^	0.159 ^a^	0.262 ^b^
Macular volume	9.915 (9.767–19.064)	9.955 (9.772–10.138)	10.026 (9.849–10.202)	0.366 ^b^	0.089 ^b^	0.277 ^b^

^a^ = Student’s t; ^b^ = Mann–Whitney U;. DM-MCI group = patients with mild cognitive impairment and diabetes. MCI group = patients with mild cognitive impairment without diabetes. C group = healthy patients.

## Data Availability

The data presented in this study are available on request from the corresponding author.
